# Salmeterol, a β2 Adrenergic Agonist, Promotes Adult Hippocampal Neurogenesis in a Region-Specific Manner

**DOI:** 10.3389/fphar.2019.01000

**Published:** 2019-09-12

**Authors:** Valeria Bortolotto, Heather Bondi, Bruna Cuccurazzu, Maurizio Rinaldi, Pier Luigi Canonico, Mariagrazia Grilli

**Affiliations:** ^1^Laboratory of Neuroplasticity, University of Piemonte Orientale, Novara, Italy; ^2^Department of Pharmaceutical Sciences, University of Piemonte Orientale, Novara, Italy

**Keywords:** adult neurogenesis, hippocampus, neural progenitor cells, beta adrenergic receptors, norepinephrine, doublecortin

## Abstract

Neurogenesis persists in the subgranular zone of the hippocampal formation in the adult mammalian brain. In this area, neural progenitor cells (NPCs) receive both permissive and instructive signals, including neurotransmitters, that allow them to generate adult-born neurons which can be functionally integrated in the preexisting circuit. Deregulation of adult hippocampal neurogenesis (ahNG) occurs in several neuropsychiatric and neurodegenerative diseases, including major depression, and represents a potential therapeutic target. Of interest, several studies suggested that, both in rodents and in humans, ahNG is increased by chronic administration of classical monoaminergic antidepressant drugs, suggesting that modulation of this process may participate to their therapeutic effects. Since the established observation that noradrenergic innervations from locus coeruleus make contact with NPC in the dentate gyrus, we investigated the role of beta adrenergic receptor (β-AR) on ahNG both *in vitro* and *in vivo*. Here we report that, *in vitro*, activation of β_2_-AR by norepinephrine and β_2_-AR agonists promotes the formation of NPC-derived mature neurons, without affecting NPC survival or differentiation toward glial lineages. Additionally, we show that a selective β_2_-AR agonist able to cross the blood–brain barrier, salmeterol, positively modulates hippocampal neuroplasticity when chronically administered in adult naïve mice. Indeed, salmeterol significantly increased number, maturation, and dendritic complexity of DCX^+^ neuroblasts. The increased number of DCX^+^ cells was not accompanied by a parallel increase in the percentage of BrdU^+^/DCX^+^ cells suggesting a potential prosurvival effect of the drug on neuroblasts. More importantly, compared to vehicle, salmeterol promoted ahNG, as demonstrated by an increase in the actual number of BrdU^+^/NeuN^+^ cells and in the percentage of BrdU^+^/NeuN^+^ cells over the total number of newly generated cells. Interestingly, salmeterol proneurogenic effects were restricted to the ventral hippocampus, an area related to emotional behavior and mood regulation. Since salmeterol is commonly used for asthma therapy in the clinical setting, its novel pharmacological property deserves to be further exploited with a particular focus on drug potential to counteract stress-induced deregulation of ahNG and depressive-like behavior.

## Introduction

Neurogenesis persists in discrete regions of adult mammalian brain. Among these regions, referred to as adult neurogenic niches, there is the subgranular zone (SGZ) in the dentate gyrus (DG) of the hippocampal formation (Ming and Song, 2011; Christian et al., 2014). In this area, adult hippocampal neurogenesis (ahNG) relies on the presence of neural progenitor cells (NPCs) which undergo proliferation and neuronal differentiation in response to instructive and permissive signals, including neurotransmitters (Eriksson et al., 1998, Spalding et al., 2013; Kempermann et al., 2015). Through a complex and highly regulated process, adult-born neuroblasts are generated and migrate to the granular cell layer (GCL) where they eventually mature into neurons which functionally integrate into the preexisting circuit (Markakis and Gage, 1999; Carlén et al., 2002; van Praag et al., 2002).

Although the physiological role of adult-born neurons is not fully understood, increasing experimental evidence suggests that they are involved in key hippocampal-dependent functions, such as learning, memory, and stress response (Snyder et al., 2011; Gonçalves et al., 2016; Anacker and Hen, 2017; Toda and Gage, 2018). In line with the idea that it represents a peculiar form of neuroplasticity, ahNG is profoundly modulated by experience and environmental conditions. Positive modulators of ahNG are environmental enrichment, physical exercise, and learning (van Praag et al., 1999; Lee et al., 2002), while relevant negative modulators are aging, stress, and social isolation (Czéh et al., 2002; Lu et al., 2003; Westenbroek et al., 2004; Bortolotto et al., 2014).

Deregulation of adult neurogenesis has been demonstrated in several neurodegenerative disorders and in neuropsychiatric diseases (as recently reviewed by Peng and Bonaguidi, 2018; Toda et al., 2019). Major depression is one of the CNS disorders where ahNG has been more extensively investigated (Malberg and Duman, 2003; Vollmayr et al., 2007). Several studies indicate that chronic administration of classical monoaminergic antidepressant drug results in enhanced hippocampal neurogenesis in the adult human and rodent DG (Santarelli et al., 2003; Boldrini et al., 2009; Perera et al., 2011; Surget et al., 2011; Boldrini et al., 2012), opening to the possibility that modulation of ahNG may contribute to the therapeutic effects of these drugs also in the clinical setting (Eisch and Petrik, 2012; Eliwa et al., 2017; Sun et al., 2017). More generally speaking, these studies suggest that ahNG can be modulated pharmacologically, and this may have therapeutic relevance in several CNS disorders.

In the last decade, our laboratory has contributed to the identification of novel molecular regulators of ahNG with potential therapeutic relevance (Denis-Donini et al., 2008; Meneghini et al., 2013; Valente et al., 2015; Cvijetic et al., 2017; Cuccurazzu et al., 2018). Through these activities, we have been able to demonstrate that several drugs utilized in the clinical setting are endowed with the ability to promote ahNG *in vitro* and, more importantly, *in vivo* (Valente et al., 2012; Cuccurazzu et al., 2013; Meneghini et al., 2014; Chiechio et al., 2017). These accomplishments have allowed us to propose novel therapeutic indications for these drugs. On the other hand, they confirm the importance of increasing our current knowledge on drugs that are already in clinic to better understand not only their effects on adult NPC but also their full profile in terms of additional mechanisms of action and/or of potential side effects/tolerability issues (Bortolotto et al., 2014; Bortolotto and Grilli, 2017; Bortolotto et al., 2017; Grilli, 2017).

Since the established observations that the hippocampus receives dense noradrenergic innervations from the locus coeruleus (LC) (Sara et al., 1994; Kitchigina et al., 1997) and that noradrenergic afferents make direct contact with proliferating cells in adult DG (Rizk et al., 2006), we decided to deeply investigate the role of beta adrenergic receptor (β-AR)–mediated effects in the adult murine hippocampus *in vitro* and *in vivo*. Herein, we show that salmeterol, a β-AR agonist commonly utilized in asthma, is a proneurogenic molecule when chronically administered in adult naïve mice. Moreover, we propose that, at least *in vitro*, distinct β-AR subtypes may mediate different effects on adult hippocampal NPCs (ahNPCs).

## Materials and Methods

**Animals.** Male C57BL/6J mice were obtained from Jackson Laboratories. β_2_-AR^−/−^ mice were kindly provided by Prof. Guido Iaccarino, Federico II University, Naples, Italy. Mice were housed in a light- and temperature-controlled room in high-efficiency particulate air (HEPA)-filtered Thoren units (Thoren Caging Systems) at the University of Piemonte Orientale animal facility. Mice were kept 3–4/cage with access to water and food *ad libitum*. Animal care and handling were performed in accordance with European Community Directive and approved by the local IACUC (Institutional Animal Care and Use Committees) Organismo Preposto al Benessere Animale (OPBA), Università del Piemonte Orientale, Novara, Italy. Only adult (3–6 months old) male mice were used for experimentation.

**Drugs.** The following drugs were utilized: L-(-)-norepinephrine-(+)-bitartrate salt monohydrate (NE) purchased from Sigma–Aldrich (Milan, Italy), salmeterol xinafoate, formoterol hemifumarate, ICI118,551 hydrochloride, CGP20712 dihydrochloride, SR59230A hydrochloride, and BRL37344 sodium salt purchased from Tocris (Bioscience, Bristol, UK). *In vitro* drug concentrations were chosen based on Ki values at their target receptors.

**Isolation and culture of adult hippocampal neural progenitor cells (ahNPCs).** For preparing NPC primary cultures from hippocampi, three adult (3–4 months old) male mice were used, and cell suspension was prepared as previously described (Valente et al., 2012). Primary (passage 1, P1) neurospheres were dissociated after 7–10 days *in vitro* (DIV), whereas P2-P30 neurospheres every five DIV. At each passage, cells were plated in T25 flask at a density of 12,000 cells/cm^2^ in complete culture medium consisting of neurobasal-A medium, supplemented with B27 supplement, 2 mM L-glutamine (Gibco, Life Technologies, Monza, IT), recombinant human epidermal growth factor (rhEGF, 20 ng/ml; PeproTech, Rock Hill, NJ), recombinant human fibroblast growth factor 2 (rhFGF-2, 10 ng/ml; PeproTech) and heparin sodium salt (4 µg/ml, Sigma–Aldrich), 100 U/ml penicillin, and 100 µg/ml streptomycin (Gibco).

**Neural progenitor cell proliferation, differentiation, and survival.** ahNPC proliferation and differentiation were assessed as previously described (Bortolotto et al., 2017). Briefly, for proliferation assays, NPCs were seeded onto 96-well plates (Falcon) at a 4,000 cells/well density in standard medium [STD medium: neurobasal-A, B27 supplement, 2 mM L-glutamine (Gibco), 10 ng/ml rhFGF-2 (PeproTech), 4 µg/ml heparin (Sigma–Aldrich), 100 U/ml penicillin, and 100 µg/ml streptomycin (Gibco)]. NPC were treated in the presence of the indicated drug concentrations or vehicle for 96 h. Proliferation medium with addition of rhEGF (20 ng/ml) was included as positive control. Proliferation rate was determined by CellTiter-Glo luminescent cell viability assay (Promega), according to manufacturer’s instructions, and standard medium values were used to normalize obtained values. In differentiation assays, NPCs were plated onto laminin-coated (2.5 µg/cm^2^) Lab-Tek 8-well Permanox chamber slides (NUNC) at the density of 43,750 cells/cm^2^ in differentiation medium [neurobasal-A medium, B27 supplement, 2 mM L-glutamine, and 100U/100 µg/ml penicillin/streptomycin (Gibco)]. NPCs were differentiated for 24 h in presence of indicated concentration of drugs or vehicle. For β-AR blockade, cells were pretreated for 30 min with selective antagonists before addition of agonist drugs. The percentage of apoptotic NPCs was evaluated after counterstaining with 0.8 ng/ml Hoechst (Thermo Fisher Scientific, Waltham, MA) diluted in PBS. Apoptotic nuclei were counted in drug- or vehicle-treated cells using a fluorescence microscope DMIRB (Leica, Wetzlar, Germany) with a 60X objective (Meneghini et al., 2010). All *in vitro* experiments were run in triplicates using different cell preparations and repeated at least three times.

**Immunocytochemical analysis.** After 24 h of differentiation, ahNPCs were fixed by 4% paraformaldehyde/4% sucrose solution and processed for immunostaining as previously described (Meneghini et al., 2014). Primary antibodies were as follows: anti-nestin (chicken monoclonal, 1:1,500, Neuromics, Edina, MN), anti-microtubule-associated protein-2 (MAP-2, rabbit polyclonal, 1: 600, Millipore, Milan, Italy), anti-glial fibrillary acidic protein (GFAP, mouse polyclonal, 1:600, Millipore), and anti-chondroitin sulfate proteoglycan (NG-2, rabbit polyclonal, 1:500, Millipore). Secondary antibodies were as follows: Alexa Fluor 488–conjugated goat anti-chicken (1:1,600), Alexa Fluor 555–conjugated goat anti-rabbit (1:1,400), Alexa Fluor 555–conjugated goat anti-mouse (1:1,600), and Alexa Fluor 488–conjugated goat anti-rabbit (1:1,400) (all from Molecular Probes, Life Technologies). Nuclei were counterstained with 0.8 ng/ml Hoechst (Thermo Fisher Scientific) diluted in PBS. In each experiment, five fields/well (corresponding to about 150–200 cells/well) were counted using the fluorescence microscope DMIRB (Leica) with a 60X objective. Immunopositive cells for each marker were counted, and their percentage over total viable cells was calculated.

***In vivo* experiments.** Adult male mice (4–6 month-old) were randomly distributed into vehicle and salmeterol treatment groups (n = 5/6). Vehicle (saline) and salmeterol (10 μg/kg body weight) were injected subcutaneously (s.c., 5 μl/g body weight) for a period of 21 days. During the first 5 days of the treatment, mice were also intraperitoneally (i.p.) injected once a day with bromodeoxyuridine (BrdU; 150 mg/kg body weight, 5 μl/g body weight, Sigma–Aldrich). Twenty-one days after the last BrdU injection, mice were deeply anesthetized and transcardially perfused with saline and then with paraformaldehyde 4% (PFA) in 0.1 M phosphate buffer, pH 7.4. After perfusion, brains were removed, postfixed in PFA 4% for 24 h, and then immersed in sucrose 30% for 24 h. In the end, brains were cut in 40 µm-thick coronal sections and stored in cryoprotectant solution at −20°C (Cuccurazzu et al., 2018).

**Immunohistochemistry.** For doublecortin (DCX) staining, procedure was as previously described (Dellarole and Grilli, 2008). Briefly, sections were incubated with goat anti-DCX primary antibody (1:1,000, Santa Cruz Biotechnology, Santa Cruz, CA) followed by biotinylated horse anti-goat secondary antibody (1:200, Vector Laboratories, Burlingame, CA). Labeled cells were visualized using the ABC system (VECTASTAIN Elite, Vector Laboratories) with 3,3’-diaminobenzidine as chromogen, and sections were then counterstained with hematoxylin (Vector Laboratories). For triple immunostaining, the following antibodies were used: rat monoclonal anti-BrdU (1:200, Novus Biologicals Inc., Littleton, CO), goat anti-glial fibrillary acidic protein (GFAP, 1:100, Santa Cruz Biotechnologies), and mouse anti-neuronal nuclei (NeuN, 1:150, Millipore). For double DCX/BrdU immunostaining, the following antibodies were used: rat monoclonal anti-BrdU (1:200, Novus Biologicals Inc., Littleton, CO) and rabbit polyclonal anti-DCX (1:200, Cell Signaling Technology Inc., Beverly, MA).

**Quantification and phenotypical characterization of newborn cells.** A modified unbiased, stereological protocol was used for quantification and phenotypic characterization of cells, as previously described (Denis-Donini et al., 2008). Briefly, a complete series of one-in-eight brain sections throughout the DG was analyzed, and an average of 8–10 sections per animal was used. The SGZ was identified as corresponding to two cell bodies wide, along the border of the GCL. For DCX analysis, positive cells were quantified using a 60X objective along the rostrocaudal extension of DG. The total number of DCX^+^ cells was obtained by adding the number of labeled cells quantified from each section and multiplying them by 8. To determine the phenotype of BrdU^+^ cells, the DG of each section was scanned by using a LSM700 confocal laser-scanning microscope (Carl Zeiss, Le Pecq, France) and a 40X/1.3 objective with a 2 µm step size. To exclude a false double labeling resulting from the overlay of signals deriving from different cells, each BrdU^+^ cell was analyzed in its entire z-axis. The absolute number of BrdU^+^, BrdU^+^/NeuN^+^, and BrdU^+^/GFAP^+^ cells was quantified in the entire DG of each section, and then numbers were summed and multiplied by 8. For the dorsal and ventral DG analysis, the anatomical coordinates were selected according to The Mouse Brain Atlas (Paxinos and Franklin, 2004). In more details, coronal brain sections located from Bregma −0.94 to −2.46 mm were considered as corresponding to dorsal DG, while those located from Bregma −2.54 to −4.04 mm were considered as corresponding to ventral DG (Elizalde et al., 2010; Lehmann et al., 2013).

In a subset of vehicle- and salmeterol-treated animals (n = 3/group), three brain sections were utilized for double DCX/BrdU immunostaining, as previously described (Meneghini et al., 2013), to evaluate the percentage of BrdU^+^ cells that had acquired a neuroblast phenotype.

**Analysis of Dendritic Morphology.** Dendritic arborization was evaluated for 70–80 DCX^+^ cells per mouse along the dorsal–ventral axis of DG. High-resolution stacks of images were obtained through a 20X/0.40 NA objective of an LSM700 laser-scanning confocal microscope (Carl Zeiss) with 0.4-µm step size. Only DCX^+^ cells within the suprapyramidal blade of DG displaying an intact dendritic arborization reaching the molecular layer (ML) and without any overlaps with other cells or artifacts were selected and three dimensionally reconstructed using the simple neurite tracer plugin (Longair et al., 2011) on the image processing package Fiji (Schindelin et al., 2012). 3D reconstructions were exported as SWC files and analyzed with L-measure tool that allowed a quantitative characterization of neuronal morphology, evaluating a wide range of morphometrical parameters (Scorcioni et al., 2008).

**Statistical analysis.** Data were expressed as mean ± S.D. and analyzed using Student’s *t*-test when only two independent groups were compared, or by one-way analysis of variance (ANOVA) followed by Tukey’s *post hoc* test when three or more groups were compared. Statistical significance level was set for p values < 0.05. For the statistical analysis of morphological parameters, a linear mixed-effects model was applied to our dataset. The mixed-effects model takes into account that several measurements were obtained from the same animal and that these measurements are expected to be correlated among themselves. In order to overcome the dependency of the repeated measurements, a random animal effect has been included in the model. The presence of significant differences was tested using one-way ANOVA for each morphological parameter. The analysis was performed in R environment implemented with the lme4 package (Bates et al., 2015). Statistical significance level was set for p values < 0.05.

## Results

### Norepinephrine (NE) Promotes Neuronal Differentiation of Adult Hippocampal Neural Progenitor Cells (ahNPCs)

**Figure 1:**
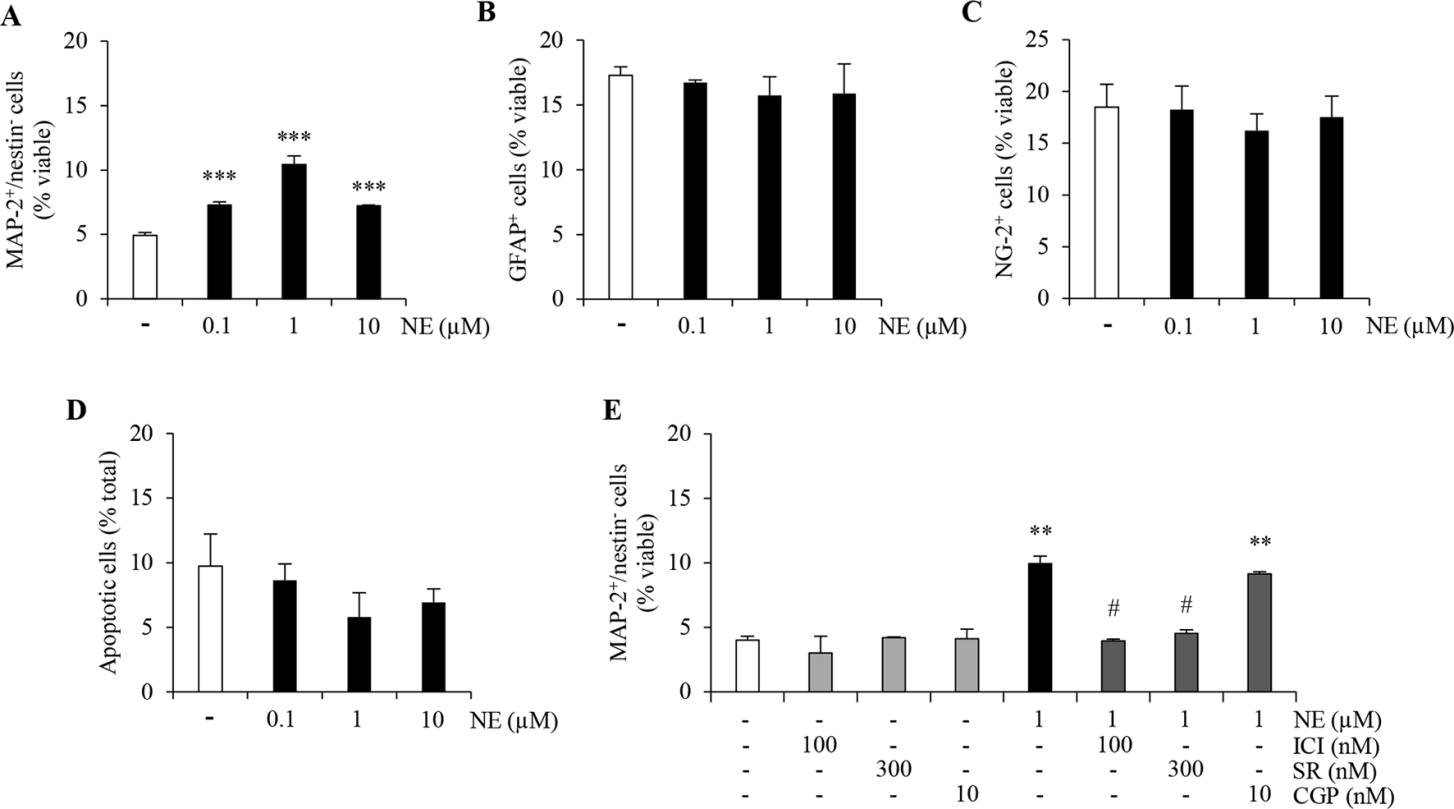
Norepinephrine (NE) promotes neuronal differentiation and proliferation of ahNPCs *via* distinct receptor subtypes. Adult hippocampal NPCs were differentiated in presence of NE (0.1–10 µM) or vehicle (−) for 24 h. The percentages of MAP-2^+^/nestin^−^ **(A)**, GFAP^+^ **(B)**, NG-2^+^ **(C)**, and apoptotic **(D)** cells were evaluated. **(E)** Pharmacological characterization of β-ARs involved in NE-mediated effects on neuronal differentiation was performed by using selective receptor antagonists: ICI118,551 (ICI, β_2_-AR antagonist), SR59230A (SR, β_3_-AR antagonist), and CGP20712 (CGP, β_1_-AR antagonist). All experiments (n = 3) were run in triplicates. Data are expressed as mean ± S.D. **p < 0.01, ***p < 0.001 *vs.* vehicle-treated cells; ^#^*p* < 0.05 *versus* NE-treated cells (one-way ANOVA, Tukey’s *post hoc* analysis).

Multipotent nestin^+^ and sox2^+^ NPCs isolated from adult mouse hippocampi can be maintained for several passages in an undifferentiated proliferative state (Cuccurazzu et al., 2013). Upon removal of growth factors followed by exposure to a serum-free defined medium, ahNPCs stop dividing and differentiating onto laminin-coated dishes. By double immunolabeling for markers of neurons (MAP-2) and undifferentiated progenitors (nestin), the appearance of newly generated MAP-2^+^/nestin^−^ neurons can be evaluated and quantified as previously described (Meneghini et al., 2013). Under these experimental conditions, norepinephrine (0.1–10 µM; NE) treatment promoted a significant proneurogenic effect as demonstrated by an increase in the percentage of MAP-2^+^/nestin^−^ cells (**Figure 1A**; p < 0.001 *versus* vehicle-treated cells, ANOVA). In absence of growth factors, ahNPCs differentiate not only toward neuronal but also glial lineages. We assessed the percentage of newly generated astrocytes and oligodendrocyte precursors, GFAP^+^ and NG-2^+^ cells, respectively. Both populations were not affected by NE treatment as shown in **Figure 1B, C** (ANOVA). Finally, no significant difference in the percentage of apoptotic cells could be observed between vehicle- and NE-treated cells (**Figure 1D**; ANOVA). Altogether, these data confirm that, *in vitro*, NE promotes neuronal differentiation of ahNPCs in absence of changes in glial differentiation and survival rate.

### NE Proneurogenic Effects Are Mediated by β_2_ and β_3_ Adrenergic Receptors

In order to pharmacologically characterize the proneurogenic effects induced by NE in our culture system, we exposed ahNPCs to the maximally effective concentration of NE (1 µM), after pretreatment with vehicle or ICI118,551 (100 nM), SR59230A (300 nM), and CGP20712 (10 nM) as selective β_2_-, β_3_-, and β_1_-AR antagonists, respectively. Both ICI118,551 and SR59230A antagonists prevented the increase in MAP-2^+^/nestin^−^ cell percentage induced by NE (**Figure 1E**; p < 0.05 *vs.* NE-treated cells, ANOVA). Conversely, the β_1_-AR antagonist CGP20712 did not affect the proneurogenic effect of NE (**Figure 1E**; ANOVA). These data suggest that, *in vitro*, NE promotes neuronal differentiation of ahNPCs *via* β_2_- and β_3_-AR.

### NE Proliferative Effects on ahNPCs Are Mediated by β_1_ Adrenergic Receptors

**Figure 2:**
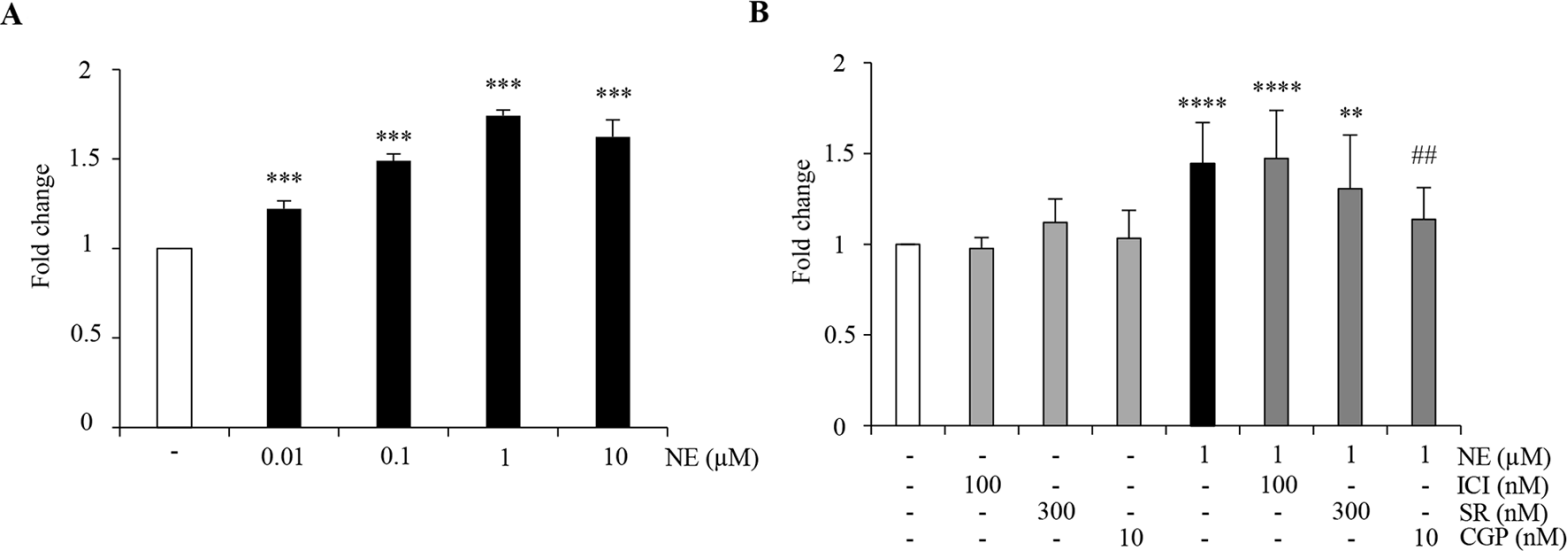
NE promotes ahNPC proliferation *via* β_1_-AR. **(A)** Evaluation of 96 h NE treatment on ahNPC proliferation. **(B)** Effects of ICI118,551, SR59230A, and CGP20712 on NE-mediated increase in ahNPC proliferation. Fold change compared to vehicle-treated ahNPCs (−). All experiments (n = 3) were run in triplicates. Data are expressed as mean ± S.D. **p < 0.01, ***p < 0.001, ****p < 0.0001 *versus* vehicle-treated cells; ^##^*p* < 0.01 *versus* 1 µM NE-treated cells (one-way ANOVA, Tukey’s *post hoc* analysis).

The above findings prompted us to investigate if distinct β-ARs may also differently modulate ahNPC proliferation. ahNPCs were seeded for 96 h in presence of NE (0.01–10 µM) and of ICI118,551 (100 nM), SR59230A (300 nM), CGP20712 (10 nM) antagonists. As shown in **Figure 2A**, NE significantly promoted ahNPC proliferation compared to vehicle-treated cells (p < 0.001, ANOVA). CGP20712 significantly counteracted NE-mediated proliferation of ahNPCs (**Figure 2B**, p < 0.01 *vs.* NE-treated cells, ANOVA), while ICI118,551 and SR59230A were ineffective (**Figure 2B**; ANOVA). These findings suggest that NE exerts a positive effect on ahNPC proliferation through activation of β_1_-AR.

### β_2_-Adrenergic Receptor Stimulation Promotes Neurogenesis *In Vitro*

**Figure 3:**
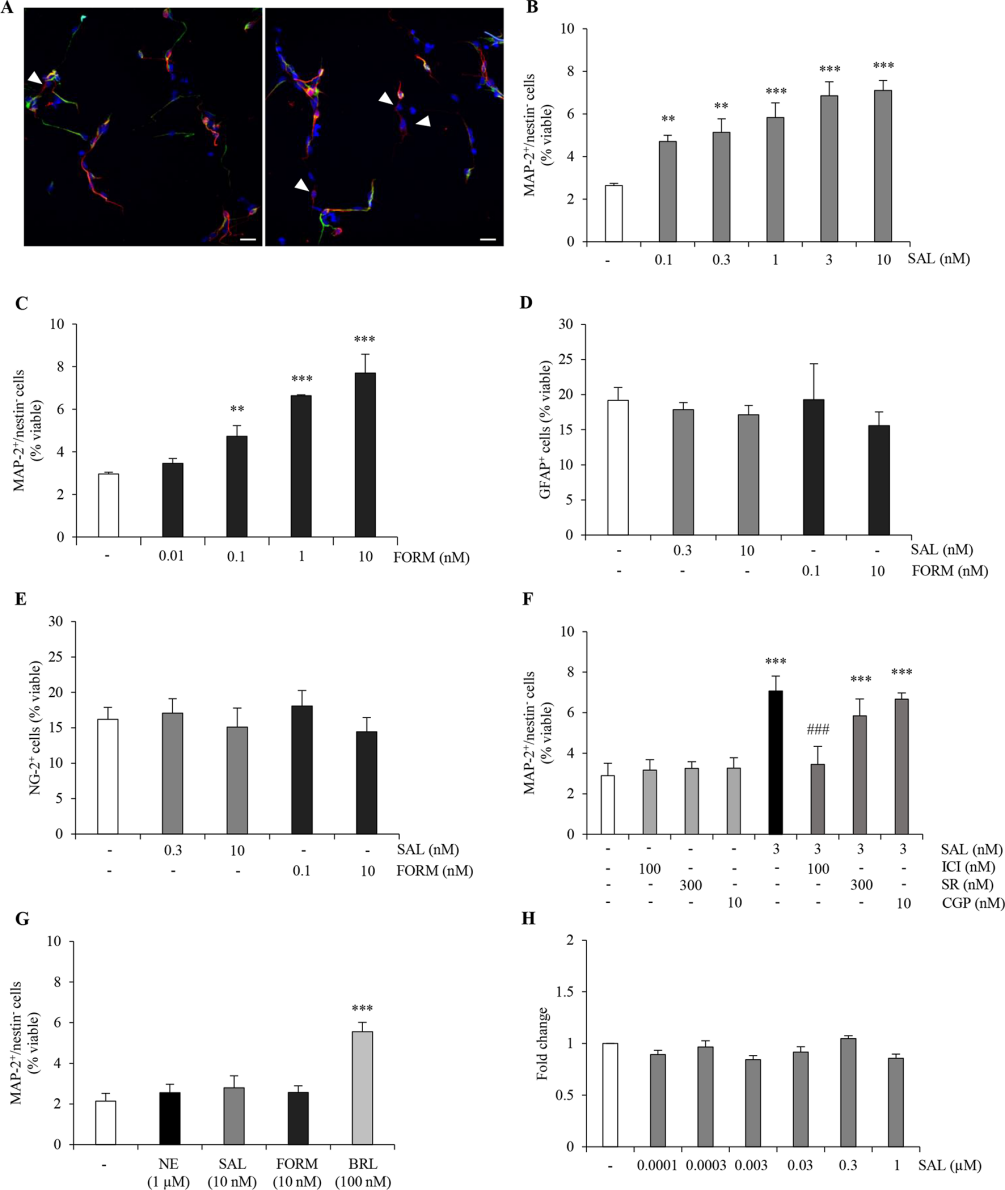
β_2_-AR agonists exert proneurogenic effects on ahNPCs. **(A)** Representative confocal images of MAP-2 (red) and nestin (green) immunolabeling in ahNPCs after 24 h treatment with vehicle (left panel) and 10 nM salmeterol (right panel). Nuclei were stained with DAPI (blue). MAP-2^+^/nestin^−^ cells are indicated (arrowheads). Magnification 400X, scale bar = 20 µm. **(B-C)** Effects of β_2_-AR agonists salmeterol (0.1–10 nM) **(B)** and formoterol (0.01–10 nM) **(C)** on neuronal differentiation of ahNPCs. **(D-E)** Effects of 24-h treatment with salmeterol (SAL) and formoterol (FORM) on GFAP^+^ **(D)** and NG-2^+^ **(E)** cells. **(F)** Effect of 3 nM SAL in presence of vehicle, ICI118551, SR59230A, or CGP20712 antagonists. **(G)** Effects of 1 µM NE, 10 nM SAL, 10 nM FORM, and 100 nM BRL37344 (BRL) on neuronal differentiation of ahNPCs derived from β_2_-AR knockout mice. **(H)** Effects of salmeterol (0.0001–1 µM) on ahNPC proliferation. Fold change compared to vehicle-treated (−) ahNPCs. All experiments (n = 3) were run in triplicates. Data are expressed as mean ± S.D. **p < 0.01, ***p < 0.001 *vs.* vehicle-treated cells; ^###^*p* < 0.001 *vs.* 3-nM SAL-treated cells (one-way ANOVA, Tukey’s *post hoc* analysis).

Previous work in our laboratory proposed that tapentadol, an analgesic drug which has low affinity for the µ-opioid receptor, has no negative effects on ahNG because of its noradrenergic mechanism, and in particular *via* its ability to activate β_2_-AR (Meneghini et al., 2014). After confirmation of β_2_-AR mRNA expression in our cellular model (*data not shown*), we extended our investigation on the role of β_2_-AR in the promotion of neuronal differentiation. Thus, we treated ahNPCs with two selective long-acting β_2_-AR agonists, salmeterol and formoterol, which are commonly utilized in asthma. In presence of salmeterol (0.1–10 nM), we observed a significant increase in the percentage of MAP-2^+^/nestin^−^ cells (**Figures 3A, B**, ANOVA). Similar effects were obtained in presence of formoterol (0.01–10 nM; **Figure 3C**). Additionally, both salmeterol (0.3–10 nM) and formoterol (0.1–10 nM) did not affect the percentage of newly generated GFAP^+^ and NG-2^+^ cells (**Figures 3D, E**; ANOVA). To confirm that salmeterol promotes neuronal differentiation of ahNPCs *via* activation of β_2_-AR, we pretreated cells with ICI118,551 (100 nM), SR59230A (300 nM), and CGP20712 (10 nM). As expected, only ICI118,551 completely prevented the proneurogenic effects induced by salmeterol (p < 0.001 *versus* salmeterol-treated cells, ANOVA), while SR59230A and CGP20712 had no effect (**Figure 3F**; ANOVA).

We also generated ahNPC cultures from β_2_-AR knockout (β_2_-AR^−/−^) mice. No difference was observed in the proliferation and differentiation rates of NPCs derived from β_2_-AR^−/−^*versus* ahNPCs derived from wild-type mice (*data not shown*). When β_2_-AR KO-derived ahNPCs were treated with salmeterol and formoterol, no proneurogenic effects were observed (**Figure 3G**, ANOVA). As a control, the β_3_-AR agonist BRL37344 could still promote neuronal differentiation of β_2_-AR KO (**Figure 3G**; ANOVA) and WT ahNPCs (*data not shown*). Altogether, these data confirmed that salmeterol and formoterol proneurogenic effects are mediated by engagement of β_2_-AR on adult hippocampal NPCs. Finally, we investigated the involvement of β_2_-AR in ahNPC proliferation. As shown in **Figure 3H**, 96 h treatment with salmeterol (0.0001–1 µM) did not change the proliferation rate of ahNPCs. Altogether, these data strongly corroborated the idea that stimulation of β_2_ adrenergic receptor results in neuronal differentiation of ahNPCs.

### Effects of Chronic Salmeterol Treatment on Number, Orientation, and Dendritic Length/Complexity of Hippocampal DCX^+^ Neuroblasts

**Figure 4:**
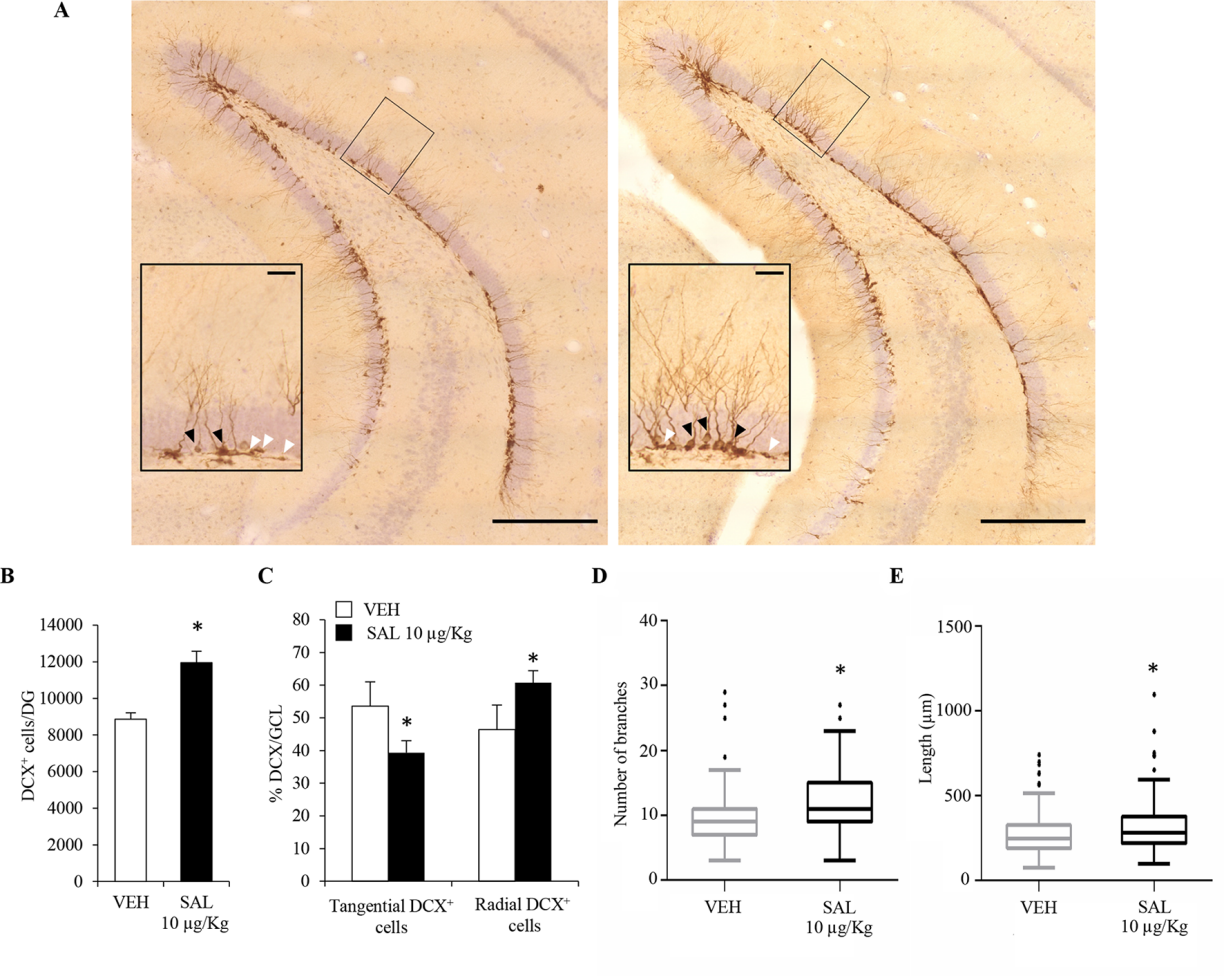
Chronic *in vivo* administration of salmeterol promotes significant changes in the number, orientation, and morphological complexity of DCX^+^ neuroblasts. **(A)** Representative photomicrographs of the DG of a mouse treated with vehicle (left panel) and salmeterol 10 µg/kg (right panel) and labeled with a DCX antibody. (Scale bar = 500 µm). The black rectangles indicate the area that is magnified as inset (scale bar = 50 µm). Higher magnification better identifies tangential (white arrowheads) and radial (black arrowheads) DCX^+^ cells in the granular cell layer. **(B)** Effect of salmeterol 10 µg/kg on the number of DCX^+^ cells in the DG. **(C)** Effects of salmeterol on the orientation of DCX^+^ cells in granular cell layer (GCL). Data are expressed as mean ± S.D.: *p < 0.05 *vs.* vehicle-treated mice (Student’s *t*-test). **(D-E)** Morphometrical analysis of DCX^+^ neuroblasts. Effects of 10 µg/kg salmeterol (SAL) on branch number **(D)** and on the arborization length **(E)** of DCX^+^ cells. Data are represented as Tukey box plots; outliers are represented by dots: *p < 0.05 *vs.* vehicle-treated mice (one-way ANOVA).

In order to understand whether chronic β_2_-AR stimulation had any effect on hippocampal plasticity *in vivo*, we performed a chronic treatment with the long-acting β_2_-AR agonist, salmeterol, which has been shown to cross the blood–brain barrier (Manchee et al., 1993). The choice of salmeterol was based on the *in vivo* potency of the drug (Qian et al., 2011). For such studies, adult male C57BL/6J mice (*n* = 5/6) received subcutaneous injections of vehicle (saline) or salmeterol (10 μg/kg) for 21 days. We initially focused our attention on newly born postmitotic neuroblasts which express the microtubule-associated protein DCX (Dellarole and Grilli, 2008). We quantified the number of DCX immunolabelled cells (**Figure 4A**) in the DG. We observed a significant increase in the number of DCX^+^ cells of salmeterol – compared to vehicle-treated mice (**Figure 4B**; vehicle: 8.9 ± 1.6 × 10^3^; salmeterol: 12.0 ± 1.6 × 10^3^, p < 0.05, Student’s *t*-test). DCX^+^ neuroblasts can be subdivided in accordance with their cell body orientation, either tangential or radial, within the GCL. Neuroblasts characterized by tangential and radial orientation are proposed to be in an initial and advanced, respectively, stage of maturation (Brown et al., 2003). Interestingly, salmeterol significantly reduced the percentage of tangentially oriented DCX^+^ cells in GCL (**Figure 4C**; tangential DCX^+^: vehicle 53.6 ± 7.4%, salmeterol 39.3 ± 3.7%, p < 0.05, Student’s *t*-test) and, in parallel, induced a significant increase in the percentage of DCX^+^ cells with radially oriented cell bodies (**Figure 4C**; radial DCX^+^: vehicle 46.4 ± 7.4%, salmeterol 60.7 ± 3.7%, p < 0.05, Student’s *t*-test). Altogether, these data corroborate the idea that *in vivo* stimulation of β_2_ adrenergic receptors results in neuroplasticity within the hippocampal DG, eliciting an increase in the number of neuroblasts and promoting their radial orientation.

To further investigate the effect of salmeterol on the maturation of neuroblasts, we also analyzed dendritic morphology and complexity in DCX^+^ cells. For each animal, 70–80 DCX^+^ cells were tridimensionally reconstructed and morphometrically characterized. Since we had multiple measurements made on multiple neurons per mouse, we used a mixed-effects approach to analyze morphometric data. By this choice, we could more accurately model our dataset in comparison to simple linear models. By morphometric analysis, we observed an overall increase of dendrite arborization induced by salmeterol. Indeed, in salmeterol-treated mice, DCX^+^ cells had a higher number of branches compared to vehicle-treated animals (**Figure 4D**; number of branches, vehicle: 9.3 ± 0.43, salmeterol: 11.6 ± 0.42, p < 0.05, ANOVA). As shown in **Figure 4E**, the total length of dendritic arborizations was also increased by chronic drug treatment (vehicle: 276.2 ± 10.8 µm, salmeterol: 319.5 ± 12.5 µm, p < 0.05, ANOVA).

### Chronic Salmeterol Administration Promotes Hippocampal Neurogenesis *In Vivo*

**Figure 5:**
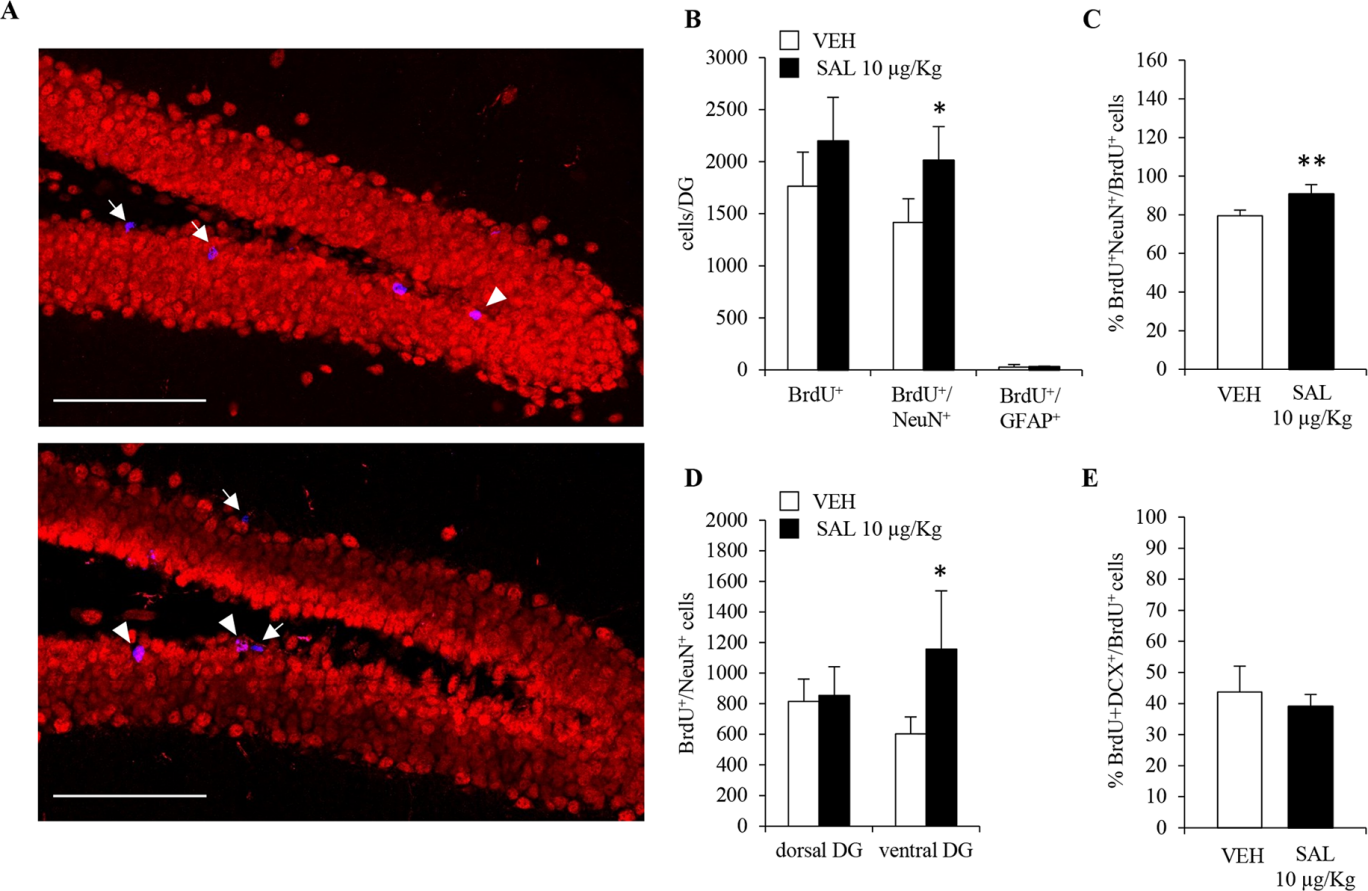
Chronic *in vivo* administration of salmeterol promotes neurogenesis in ventral hippocampus. **(A)** Representative confocal microscopic images of BrdU (blue), NeuN (red), immunolabeling in hippocampi of vehicle-treated (upper panel), and 10 µg/kg salmeterol-treated mice (lower panel). Single-positive BrdU cells (arrows) and BrdU^+^/NeuN^+^ cells (arrowheads) in the murine DG are indicated at 40X magnification (scale bar = 100 µm). **(B)** Quantitative analysis of BrdU^+^, BrdU^+^/NeuN^+^, and BrdU^+^/GFAP^+^ cells in DG of vehicle- and 10 µg/kg salmeterol-treated mice. **(C)** Quantitative analysis of the percentage of newly generated neurons (BrdU^+^/NeuN^+^ cells) over total number of BrdU^+^ cells in DG of vehicle- and 10 µg/kg salmeterol-treated mice. **(D)** Quantitative analysis of BrdU^+^/NeuN^+^ cells in dorsal and ventral DG of vehicle- and 10 µg/kg salmeterol-treated mice. **(E)** Quantitative analysis of the percentage of BrdU^+^ cells that acquired DCX^+^ phenotype in DG of vehicle- and 10 µg/kg salmeterol-treated mice. Data are expressed as mean ± S.D.: *p < 0.05, **p < 0.01 *vs.* vehicle-treated mice (Student’s *t*-test).

Despite changes in number, orientation and morphology of DCX^+^ neuroblasts in salmeterol-treated mice, a *bonafide* increase in adult hippocampal neurogenesis still needed to be rigorously addressed. Indeed, the increased number of neuroblasts in salmeterol-treated mice could also be due to drug-induced prosurvival effects. To this end, we took advantage of the fact that mice, chronically administered with salmeterol or vehicle, also received the thymidine analog BrdU (150 mg/kg of body weight, i.p.) during the first 5 days of treatment. The total number of BrdU^+^ cells was quantified in the DG of both vehicle- and salmeterol-treated mice, and no significant differences could be observed between the two groups (**Figures 5A, B**; vehicle: 1.7 ± 0.3 X 10^3^, salmeterol: 2.2 ± 0.4 X 10^3^; p = 0.13, Student’s *t*-test). When we compared the number of BrdU^+^/NeuN^+^ cells, we observed a significant increase in mice treated with salmeterol compared with vehicle animals (**Figure 5B**; vehicle: 1.4 ± 0.2 X 10^3^, salmeterol: 2.0 ± 0.3 X 10^3^; p < 0.05, Student’s *t*-test). We also calculated the percentage of BrdU^+^/NeuN^+^ cells over the total number of BrdU^+^ cells in the two experimental groups (**Figure 5C**). We confirmed an increased percentage of newly generated neurons in salmeterol mice (**Figure 5C**; % BrdU^+^NeuN^+^/BrdU^+^ cells: 79.4 ± 2.9 and 90.7 ± 4.9 in vehicle *versus* salmeterol-treated mice, respectively; p < 0.01, Student’s *t*-test). In agreement with *in vitro* data, no difference was observed in the number of BrdU^+^/GFAP^+^ cells between the two experimental groups (**Figure 5B**; vehicle: 26 ± 25.6, salmeterol: 30 ± 7.6, Student’s *t*-test). Interestingly, when we examined dorsal and ventral hippocampi separately, we highlighted region-specific effects elicited by salmeterol. In the ventral DG of drug-treated mice, the number of BrdU^+^/NeuN^+^ cells was significantly increased compared to vehicle-treated group (**Figure 5D**; vehicle: 602 ± 111.7, salmeterol: 1158 ± 380.2, p < 0.05; Student’s *t*-test). Conversely, no significant difference between vehicle- and drug-treated mice could be observed in the dorsal DG (**Figure 5D**; vehicle: 816 ± 146.3, salmeterol: 854 ± 187.4; Student’s *t*-test.). Altogether, these data demonstrate that chronic salmeterol treatment promotes adult hippocampal neurogenesis *in vivo*, and, more specifically, in the ventral DG. In order to better understand the dynamic scenario occurring between neuroblasts and mature neuron formation in response to salmeterol, we also performed a double BrdU/DCX immunolabeling and calculated the percentage of hippocampal BrdU^+^ that had acquired a DCX^+^ phenotype. Surprisingly, we did not observe a significant difference between vehicle- and salmeterol-treated animals (**Figure 5E**; % BrdU^+^DCX^+^/BrdU^+^ cells: 43.7 ± 8.3 and 39.1 ± 3.9 in vehicle *versus* drug-treated mice, respectively; p = 0.43, Student’s *t*-test). Since the overall number of BrdU^+^ cells is not statistically different between experimental groups, although there was a trend increase in drug- *versus* vehicle-treated mice, we hypothesize that the increase in the number of DCX^+^ cells observed in the salmeterol group could be due to an increased survival of hippocampal neuroblasts.

## Discussion

The SGZ of the hippocampal DG is one of the brain regions where generation of new neurons occurs throughout life (Altman and Das, 1965; Cameron et al., 1993; Eriksson et al., 1998; Gould et al., 1998). In this region, adult NPCs proliferate and give rise to neuroblasts migrating to the GCL where they mature into neurons which may functionally integrate into the preexisting circuit (Markakis and Gage, 1999; Carlén et al., 2002; van Praag et al., 2002).

A vast array of signals affects and modulates ahNG, including neurotransmitter activity (Hagg, 2007; Toda et al., 2019). The dense monoaminergic innervation of the DG has attracted great interest for its contribution to adult neurogenesis modulation. At least in part, this is due to the fact that ahNG is proposed to underlie some of the behavioral effects elicited by classical antidepressants (Santarelli et al., 2003; Warner-Schmidt and Duman, 2006; Eliwa et al., 2017; Sun et al., 2017), whose actions are triggered by increased extracellular levels of serotonin and/or norepinephrine. Extensive research efforts have dissected the role of serotonin receptor subtypes in the regulation of ahNG both *in vitro* and *in vivo* (Alenina and Klempin, 2015; Bortolotto et al., 2017). Comparatively, less is known about the role of adrenergic receptor subtypes in ahNG.

The noradrenergic transmission plays key regulatory roles in a variety of physiological processes, including specific aspects of learning and memory which have been functionally correlated with ahNG (Amaral and Sinnamon, 1977; Aston-Jones and Cohen, 2005; Benarroch, 2009; Sara, 2009). Notably, the hippocampus is one of the brain areas receiving the densest noradrenergic innervation from LC (Swanson and Hartman, 1975), and LC-derived noradrenergic afferents make direct contact with proliferating cells in adult DG (Rizk et al., 2006). In previous work, several groups have reported a permissive role for NE on adult hippocampal neurogenesis (Kulkarni et al., 2002; Jhaveri et al., 2010; Coradazzi et al., 2016). As far as receptor subtypes, while no major role of α_1_-AR has been proposed, α_2_-AR was initially suggested to be involved in the regulation of ahNG. Indeed, chronic treatment of rats with dexefaroxan, an α_2_-AR antagonist, promoted long-term survival of newborn hippocampal neurons by enhancing the number and complexity of the dendritic arborizations of PSA-NCAM neurons, potentially *via* BDNF (Rizk et al., 2006). Less is known about the contribution of β-ARs, which are expressed in the hippocampus (Nicholas et al., 1993) and involved in learning and memory (Sara et al., 1994), in the modulation of hippocampal neurogenesis. Jhaveri et al., (2014) suggested that a balance between α_2_- and β-AR activity may regulate NPC activity and hippocampal neurogenesis. They showed that selective stimulation of α_2_ adrenergic receptors decreases NPC proliferation and immature neuron number, while stimulation of β adrenergic receptors activates the quiescent precursor pool and enhances their proliferation in the adult hippocampus. In their study, the authors used, as pharmacological tools, isoproterenol and propranolol as nonselective β-AR agonist and antagonist, respectively. Based on previous work showing that the β_3_-AR agonist BRL37344 had proliferative effects in the neurosphere assay (Jhaveri et al., 2010), the authors suggested that the *in vitro* effects of isoproterenol and NE could also be mediated by β_3_-ARs. On the other hand Masuda et al. (2012) by using adult rat NPCs suggested that NE pro-proliferative effects were mediated by β_2_-AR.

Based on these premises, we decided to further dissect the role of distinct β-AR in NE effects in adult hippocampal neurogenesis. By extensive pharmacological characterization, we initially proved that adult hippocampal NPCs functionally express β_1/2/3_-AR and that each receptor subtype exerts distinct effects when stimulated by NE, despite the fact that they all increase intracellular cAMP concentrations. We demonstrated that NE is able to significantly increase ahNPC proliferation *in vitro* and that this effect appears mediated by the β_1_-AR subtype. Indeed, a selective β_1_-AR antagonist completely inhibited NE-mediated NPC proliferation, while selective β_2_- and β_3_-AR antagonists were ineffective. Moreover, again by using selective antagonists, we demonstrated that β_2_/β_3_-AR activation–mediated NE effects on neuronal differentiation of ahNPCs. The contribution of β_2_-AR to neurogenesis was further confirmed by the fact that NE and the β_2_-AR agonists salmeterol and formoterol were ineffective in promoting neuronal differentiation of primary cultures of ahNPCs derived from β_2_-AR^−/−^ mice. Interestingly, stimulation of β_3_-AR could still promote neurogenesis in β_2_-AR^−/−^ NPC cultures, confirming an additional role of this receptor subtype, as previously suggested (Jhaveri et al., 2010; Jhaveri et al., 2014).

These experimental observations prompted us to test clinically relevant β_2_-AR agonists, commonly utilized antiasthmatic drugs, as additional pharmacological tools for *in vivo* studies. Since not all β_2_-AR agonists cross the blood–brain barrier, for such studies, we selected salmeterol, a long-acting β_2_-AR. The drug, when administered subcutaneously for 21 days, was able to significantly promote neurogenesis in the adult murine hippocampus, as revealed by an increase in the number of BrdU^+^/NeuN^+^ newly generated neurons, as well as by a statistically significant increase in the percentage of BrdU^+^NeuN^+^/BrdU^+^ cells compared to vehicle-treated animals. In agreement with what we observed *in vitro* in adult hippocampal NPC cultures, salmeterol administration had no significant effect on astrogliogenesis *in vivo*, since no difference was reported in the number of BrdU^+^/GFAP^+^ cells when compared with vehicle-treated mice. At least *in vitro*, we could exclude any effect of NE and β_2_-AR agonists not only on astrogliogenesis but also on the number of NG-2^+^ oligodendrocyte precursors which are generated by ahNPCs.

The proneurogenic effects of chronic salmeterol administration were accompanied also by a significant increase in the actual number of DCX^+^ cell in the DG of salmeterol-treated mice. DCX is a microtubule-associated protein which is expressed by immature neurons in the adult DG and involved in cell migration (Corbo et al., 2002; Bai et al., 2003). For these reasons, DCX is commonly utilized as a marker of adult-born neuroblasts (Dellarole and Grilli, 2008). In parallel with the increased number of DCX^+^ cell in the DG of salmeterol-treated mice, we also observed a remarkable increase in the percentage of radially compared to tangentially oriented immunopositive cells. Interestingly, radial DCX^+^ cells are considered to represent a more advanced stage of maturation and migration compared to the ones which are tangentially oriented (Gleeson et al., 1999). Due to its distribution and pattern expression, DCX is also widely used for morphometric analysis of dendritic arborizations of adult-born neuroblasts (Kempermann et al., 2003). Our analysis revealed a significant positive effect of salmeterol administration on DCX^+^ dendritic complexity and length, compared to vehicle. Together with the changes in DCX^+^ cell orientation, our data suggested the idea that chronic salmeterol treatment increases hippocampal neuroplasticity by promoting DCX^+^ cell maturation. Surprisingly, when we quantified the percentage of hippocampal BrdU^+^DCX^+^/BrdU^+^ cells, we did not observe a significant difference between vehicle- and salmeterol-treated animals. Since the overall number of BrdU^+^ cells is not statistically different between groups, although there was a trend increase in drug- *versus* vehicle-treated mice, one potential explanation is that the increased number of DCX^+^ cells in the salmeterol group is due to increased survival of hippocampal neuroblasts. These findings are particularly interesting in view of the proposed heterogeneity within the DCX population (Walker et al., 2007) and previous reports that salmeterol may exert neuroprotective effects mediated by glial cells (Qian et al., 2011). Future studies will need to be properly designed to better understand the distinct effects of salmeterol administration on maturation and/or survival of adult-born hippocampal neuroblasts and neurons. *In vitro*, we did not observe a prosurvival effect of β_2_-AR agonists on NPC neuronal progeny, so it is possible that salmeterol *in vivo* effects is mediated by cell populations which are underepresented or absent in culture.

Based on data in ahNPC cultures, salmeterol proneurogenic effects appear to be, at least in part, cell autonomous, while we cannot exclude the possibility that cell types other than ahNPCs may also contribute to drug-mediated proneurogenic and/or prosurvival effects *in vivo*. Astrocytes indeed express β_2_-AR whose stimulation can mediate release of trophic factors which, in turn, may promote hippocampal neurogenesis (Laureys et al., 2010). Activation of AR on astrocytes may also affect neurogenesis indirectly, through neuronal metabolic support by astroglia. Indeed, astroglial aerobic glycolysis is regulated by NE through β-AR/cAMP signaling (Vardjan et al., 2014).

Interestingly, the proneurogenic effect of chronic salmeterol treatment was restricted to the ventral, but not the dorsal, region of the hippocampus. At the present stage, no literature data—for example, different expression levels of β_2_-AR in ventral hippocampus (vHp) *versus* dorsal hippocampus (dHp), provide a clear explanation for region specificity in the proneurogenic effects of salmeterol, but they may deserve further investigation. Unfortunately, no reliable β_2_-AR antibodies are currently available. Despite these limitations, the finding of the region specificity of salmeterol is quite interesting since anatomical and functional segregation along the hippocampal dorso–ventral axis is a well established concept. The dHp mainly receives inputs from cortical areas, whereas the vHp is much more closely connected to subcortical structures, such as amygdala and the hypothalamic–pituitary–adrenal axis (Grilli et al., 1988; Strange et al., 2014). In line with anatomical data, dHp appears to be preferentially involved in spatial navigation/memory and learning (Kim and Fanselow, 1992; Yoon and Otto, 2007; Fanselow and Dong, 2010), while the vHp has been connected with emotional reactivity and behavior (Pentkowski et al., 2006; Goodrich-Hunsaker et al., 2008; McHugh et al., 2011). Worth of note, it has been suggested that dorsal and ventral ahNG may also be involved in different functions, with dorsal ahNG more correlated with cognitive functions while ventral ahNG with emotional behavior and mood regulation (Tanti and Belzung, 2013). In addition, external stimuli such as stress or drugs regulate adult neurogenesis differently along this axis. Previous reports have suggested that stress preferentially elicits deleterious effects on ventral hippocampal neurogenesis and that a decrease of ahNG in this area could be sufficient for the induction of depressive-like behavior (Felice et al., 2012; Hawley et al., 2012). Moreover, chronic treatment with different antidepressant drugs increased neurogenesis predominantly in vHp of rodents (Banasr et al., 2006; Wu and Hen, 2014; Zhou et al., 2016). This idea is further supported by studies in depressed patients showing prominent effects of selective serotonin reuptake inhibitors and tricyclic antidepressants in the anterior part of the hippocampus, the human correlate of rodent vHp (Boldrini et al., 2009, Boldrini et al., 2012). Interestingly, anatomical and biochemical evidence also support the idea that LC fibers in the fornix mainly innervate the dorsal DG while cingulum projects mainly to the ventral hippocampal formation where it supplies fibers to DG (Haring and Davis, 1985).

With the present work, we demonstrated hippocampal neuroplasticity and neurogenesis induced by chronic β_2_-AR agonist administration. The proneurogenic effects of salmeterol were restricted to the vHp. The behavioral correlates of these effects remain to be further investigated. Specifically, the effects of salmeterol in animal models of stress-induced depressive-like behavior could be investigated. Additionally, the possibility that β_2_-AR agonists that pass the blood–brain barrier may enhance antidepressant effects exists and could be tested. In light of the fact that a third of depressed patients do not satisfactorily respond or are resistant to antidepressant treatment, these observations certainly deserve further attention.

## Data Availability

All datasets generated for this study are included in the manuscript/supplementary files.

## Ethics Statement

The animal study was reviewed and approved by OPBA, University of Piemonte Orientale.

## Author Contributions

MG conceived research and, with VB, designed methodologies and experiments. VB, BC, and HB performed experiments and analyzed results. PLC analyzed results. MR performed statistical analysis. MG wrote the manuscript with input from coauthors. All authors participated in discussion and proofreading of the manuscript.

## Funding

This work was partially supported by PRIN MIUR and Fondazione Cariplo to MG. HB held a research fellowship (*Bando Fondazione CRT*, ID 393) supported by University of Piemonte Orientale. VB was supported by a SIF/MSD fellowship 2016.

## Conflict of Interest Statement

The authors declare that the research was conducted in the absence of any commercial or financial relationships that could be construed as a potential conflict of interest.
